# Superior Hydrogen Sensing Property of Porous NiO/SnO_2_ Nanofibers Synthesized via Carbonization

**DOI:** 10.3390/nano9091250

**Published:** 2019-09-03

**Authors:** Hongcheng Liu, Feipeng Wang, Kelin Hu, Bin Zhang, Li He, Qu Zhou

**Affiliations:** 1 State Key Laboratory of Power Transmission Equipment & System Security and New Technology, Chongqing University, Chongqing 400044, China; 2 Analytical and Testing Center of Chongqing University, Chongqing 401331, China; 3 College of Engineering and Technology, Southwest University, Chongqing 400715, China

**Keywords:** NiO/SnO_2_ nanofibers, p-n heterojunctions, porous nanostructure, H_2_ sensor

## Abstract

In this paper, the porous NiO/SnO_2_ nanofibers were synthesized via the electrospinning method along with the carbonization process. The characterization results show that the pristine SnO_2_-based nanofibers can form porous structure with different grain size by carbonization. The hydrogen gas-sensing investigations indicate that the NiO/SnO_2_ sensor exhibits more prominent sensing properties than those of pure SnO_2_ sensor devices. Such enhanced performance is mainly attributed to the porous nanostructure, which can provide large active adsorption sites for surface reaction. Moreover, the existence of p-n heterojunctions between NiO and SnO_2_ also plays a key role in enhancing gas-sensing performances. Finally, the H_2_ sensing mechanism based on the NiO/SnO_2_ nanocomposite was proposed for developing high-performance gas sensor devices.

## 1. Introduction

Hydrogen (H_2_) is a key characteristic parameter that reflects the spark, arc and partial discharge faults in transformer oil [1]. Using the gas sensor technology to realize the on-line monitoring of H_2_ gas can effectively analyze the operating state of the transformer to ensure the power supply requirements in our life [2]. In the field of gas sensors, semiconductor oxide sensors, such as ZnO [3,4], WO_3_ [5], SnO_2_ [6,7], CuO [8], NiO [9], etc., have been widely studied due to the characteristics of favorable gas sensitivity and easy synthesis, as well as the ability of detecting various gases due to the unique physical and chemical properties.

It is known to all that the intrinsic semiconductor gas-sensing materials have the disadvantages of poor gas response, high operating temperature, and long response-recovery time owing to the inherent defects of semiconductors [10,11]. Thus, a large number of researches about using various synthetic techniques to control the surface morphology of sensing materials have been reported [12,13,14]. Moreover, extensive studies have been devoted to the optimization of gas sensor performances by doping method [15,16,17,18]. For instance, Katoch et al. [19] reported that smaller hole-diameter ZnO fibers synthesized by electrospinning possess more sensitive gas response to target gases than those with larger diameters, since the ZnO fibers has an increase in the surface area. Xue et al. [20] fabricated a sensor with flower-like CeO_2_-SnO_2_ composites via hydrothermal reaction which exhibited enhanced gas response, more selective and better linearity for triethylamine gas compared to the pure SnO_2_ sensor. In particular, NiO (p-type) and SnO_2_ (n-type), two typical semiconductors with wide band gaps, which are able to form p-n heterojunctions at their interface, have attracted a lot of interest of researchers [21,22]. Jayababu et al. [23] synthesized the NiO/SnO_2_ nanocomposites by a simple two-step process (co-precipitation technique followed by sol-gel method); the sensor based on the nanocomposites shown improved ethanol gas-sensing properties in comparison with the SnO_2_ and NiO sensor devices. And these advanced sensing characteristics were chiefly due to the existence of p-n heterojunctions. Meng et al. [24] reported a gas sensor which was fabricated with the NiO-SnO_2_ heterojunction microflowers. The gas-sensing investigations exhibited that the 5 mol% NiO-doped SnO_2_ composite sample has improved sensing performances to HCHO gas. However, the synthesis of nanomaterials with large surface area and multi-active adsorption sites are still a significant but challenging task. Moreover, to the best of our knowledge, SnO_2_, ZnO, TiO_2_/SnO_2_ and In/NiO nanofiber sensitive materials and their gas-sensing performances have already been studied [25,26,27,28]. While the synthesis of porous NiO/SnO_2_ nanofibers with carbonization process in various heating rates for H_2_ gas sensors was much less widely reported.

Herein, we have successfully synthesized porous composites of SnO_2_-based nanofibers with the electrospinning method combined with the carbonization process. The as-prepared SnO_2_ sensor with various carbonization heating rates cause changes in the gas sensing performances due to the different micro grain size of the sample. The gas-sensing characteristics of SnO_2_-based nanocomposites to H_2_ (1–250 ppm) at various optimal operation temperature (165–240 °C) have been studied. The investigated results indicate that the NiO/SnO_2_ nanofibers can enhance the H_2_ gas-sensing properties to a large extent. Such remarkable characteristics can be principally due to the unique porous microstructure and the formation of p–n heterojunctions at the interface of NiO and SnO_2_.

## 2. Experimental Procedure

### 2.1. Preparation

Nickel chloride hexahydrate (NiCl_2_·6H_2_O, 99.9%), Tin chloride dehydrate (SnCl_2_·2H_2_O, ≥ 99.9%), Polyvinylpyrrolidone (PVP, Mw = 1,300,000), and N,N-Dimethylformamide (DMF, ≥ 99.9%) were obtained from Aladdin Chemical Co., Ltd. (Shanghai, China). Absolute ethanol was bought from Chongqing Chuandong Chemical Reagent Co., Ltd. (Chongqing, China). All the chemicals reagents were used as received without any further treatment.

**Figure 1:**
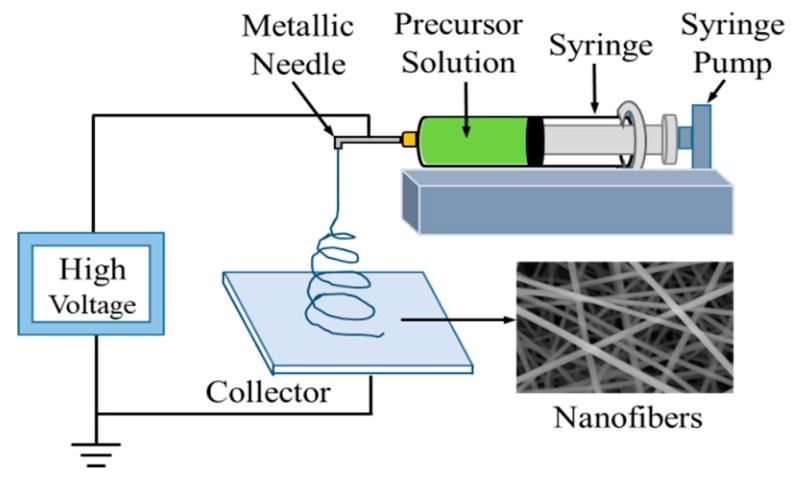
Schematic diagram of electrospinning.

Firstly, 1.128 g SnCl_2_·2H_2_O, 0.209 g NiCl_2_·6H_2_O (nickel atoms account for 15% of the total metal salt), absolute ethanol (5 mL) and DMF (5 mL) were added to a beaker (25 mL) and magnetically stirred for about 30 min until the solute dissolved completely. Then, 0.8 g PVP was added into the above solution and vigorously stirred for 6 h at 50 °C. After that, the prepared precursor solution was filled to a plastic 10 mL syringe with an inner diameter metallic needle of 0.8 mm. As shown in Figure 1, a high voltage of 12 kV was applied between the flat tin foil collector and the needle with the spacing of 15 cm. The precursor solution was continuously fed at a rate of 1 mL/h and the chamber was maintained at an appropriate environment condition of 35 °C and 45% relative humidity. Finally, the pristine electrospun nanofibers (marked as NiSn/0) were collected and dried at room temperature for 2 h to further use. For comparison, the pristine of pure SnO_2_ nanofiber, named Sn/0, was prepared by the same process without adding NiCl_2_·6H_2_O.

### 2.2. Carbonization

The prepared precursor electrospun pure SnO_2_ nanofibers were delivered to an alumina ceramic crucible and annealed at 500 °C for 3 h with diverse aging rates (2 °C/min and 4 °C/min, respectively) in air atmosphere. For convenience, we denoted the carbonated SnO_2_ products as Sn/2 and Sn/4, respectively. Moreover, the pristine NiO/SnO_2_ nanofibers were carbonized by the similar process, and annealed with aging rate of 4 °C/min in air (labeled as NiSn/4).

### 2.3. Characterization

The X-ray diffraction (XRD, Spectris Pte. Ltd. PANalytical X’Pert Powder, Almelo, Holland, operated at 40 kV and 40 mA) with Cu Kα radiation (λ = 0.15418 nm) and scanning electron microscopy (SEM, MIRA3 LMH, TESCAN, Brno, Czech, operated at 10 kV) were used to examine the crystalline parameters and microstructures, respectively. High resolution transmission electron microscopy (HRTEM) and high angle annular dark field (HAADF) images of our products were recorded by Thermo Fisher Scientific (FEI Talos F200S G2, Bleiswijk, Holland). Additionally, the chemical compositions of the samples were obtained by energy dispersive X-ray spectroscopy (EDS) elemental mapping and spot analyses. X-ray photoelectron spectroscopy (XPS, Thermo Fisher Scientific ESCALAB 250Xi) with Al Kα radiation was used to study the surface chemical state of our samples. The Brunauer-Emmett-Teller (BET) surface area and pore size were examined by the Quadrasorb 2MP analyzer.

### 2.4. Fabrication and Measurement

Gas-sensor devices based on nanofiber sensing-materials were fabricated via the same technology as the previously reported article [29]. In this study, gas sensing properties of the obtained sensor devices at different working temperatures for various concentrations of H_2_ were studied by the Chemical Gas Sensor-8 (CGS-8) intelligent gas sensing analysis system (Beijing Elite Tech Co., Ltd., Beijing, China) [30]. The sensor response was denoted by S = Ra/Rg, in which Ra is the resistance in atmospheric air, and Rg is the resistance in the target gas [31]. The response and recovery time of gas sensor devices were defined as the time required by the sensor to achieve 90% of the total resistance after injecting and removing the target gas [32]. The measurements of these sensors were all conducted in constant laboratory environmental conditions with temperature 25 °C and 50% relative humidity.

## 3. Results and Discussion

### 3.1. Structural and Morphological Characterizations

**Figure 2:**
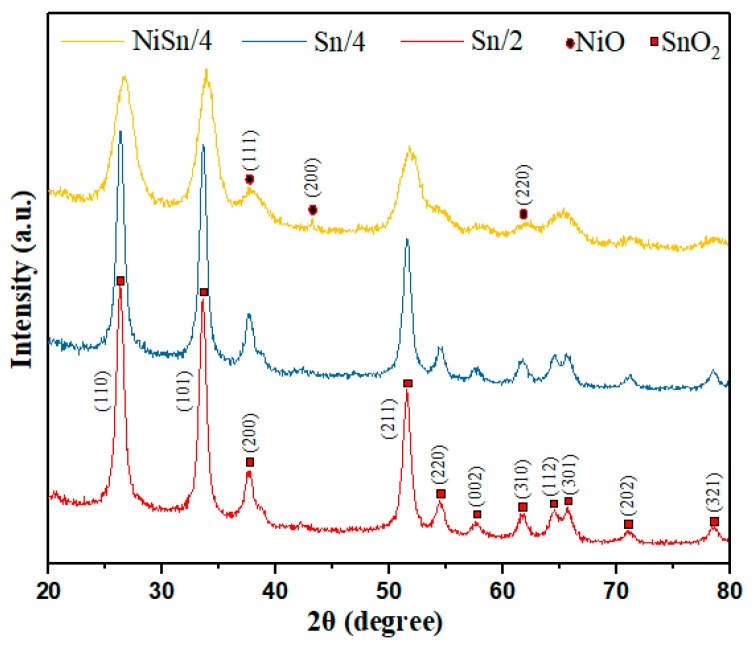
X-ray diffraction (XRD) spectra of Sn/2, Sn/4 and NiSn/4 nanofibers.

Figure 2 displays the XRD spectra of the Sn/2, Sn/4 and NiSn/4 samples. It can be seen from Figure 2 for spectrum of the Sn/2, Sn/4 products that the diffraction peaks marked with the corresponding angles can be well matched the SnO_2_ (JCPDS File NO.41-1445) [33]. The XRD spectrum of NiSn/4 nanofiber shows that the introduction of NiO broadens the diffraction peaks of the sample, indicating that the crystal lattice of the composite nanofibers may be distorted. Moreover, it is difficult to observe the diffraction peaks of NiO, which may due to its small content and the presence of the second phase in the diffraction peak of SnO_2_ [34]. The average grain sizes of the most prominent diffraction peaks of (110), (101) and (211) for the Sn/2, Sn/4 and NiSn/4 nanofibers, located at 26.72°, 34.04° and 51.81°, respectively, were calculated using the Debye-Scherer equation (D = kλ/ρcosθ). Results were approximately 12.12 nm, 11.38 nm and 6.29 nm, respectively [35].

**Figure 3:**
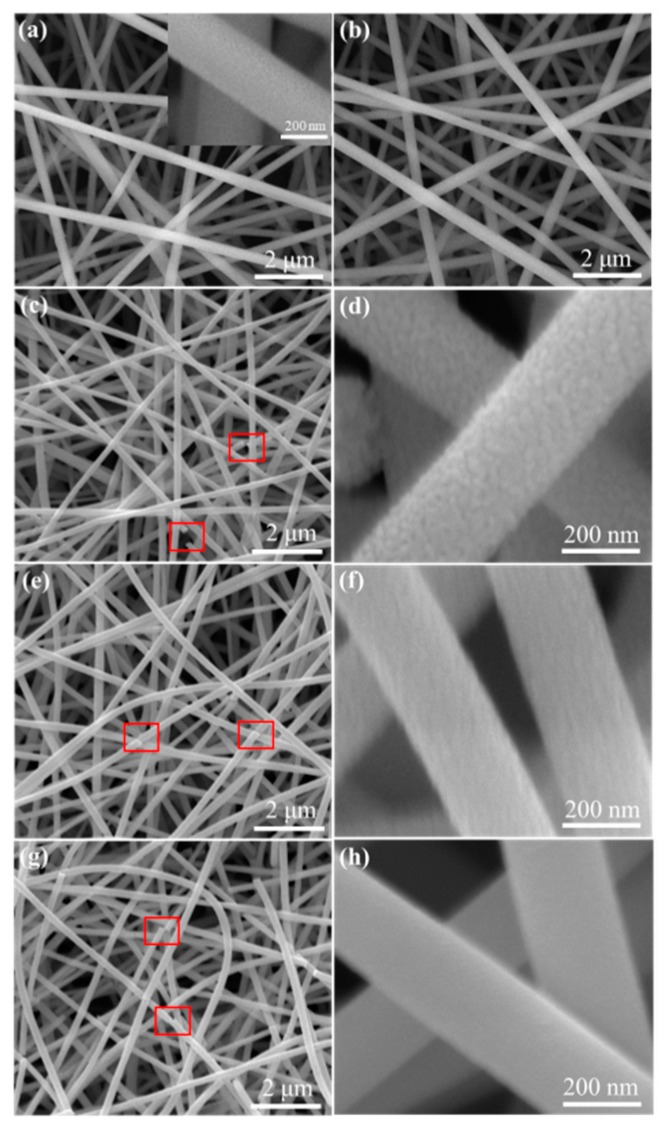
Typical scanning electron microscopy (SEM) images of (**a**) Sn/0, (**b**) NiSn/0, (**c**) Sn/2, (**e**) Sn/4 and (**g**) NiSn/4 samples. (**d**,**f**,**h**) are the relative high-resolution images of (**c**,**e**,**g**), respectively.

The surface topography characteristics of the electrospun samples were examined via SEM as shown in Figure 3. From Figure 3a,b, it can be seen that both the pristine Sn/0 and NiSn/0 samples exhibit continuous multi-layered fiber-like shapes in a random distribution. The diameter of a single nanofiber is approximately 200 nm as shown in the insert picture from Figure 3a,c,e,g present the SEM images of the carbonized SnO_2_-based nanofibers under different aging conditions. We can find that the surface morphology of the samples has hardly changed after aging, except for a few fibers fracture as indicated by the red box in the images of Figure 3c,e,g. This phenomenon may be caused by the thermal effects during the aging process [36]. Figure 3d,f,h show high-magnification SEM images of Sn/2, Sn/4 and NiSn/4 nanofibers. Obviously, the surface topography of the electrospun samples became rough due to the thermo decomposition of PVP [37]. In addition, the carbonized pure SnO_2_ nanofibers as displayed in Figure 3d,f show different surface roughness at different heating rates. The surface of the Sn/2 nanofiber obtained by slow heating rate is rougher than the Sn/4 sample. The surface of the composite NiSn/4 nanofiers as shown in Figure 3h was relatively smooth, which may attribute to the introduction of NiO.

**Figure 4:**
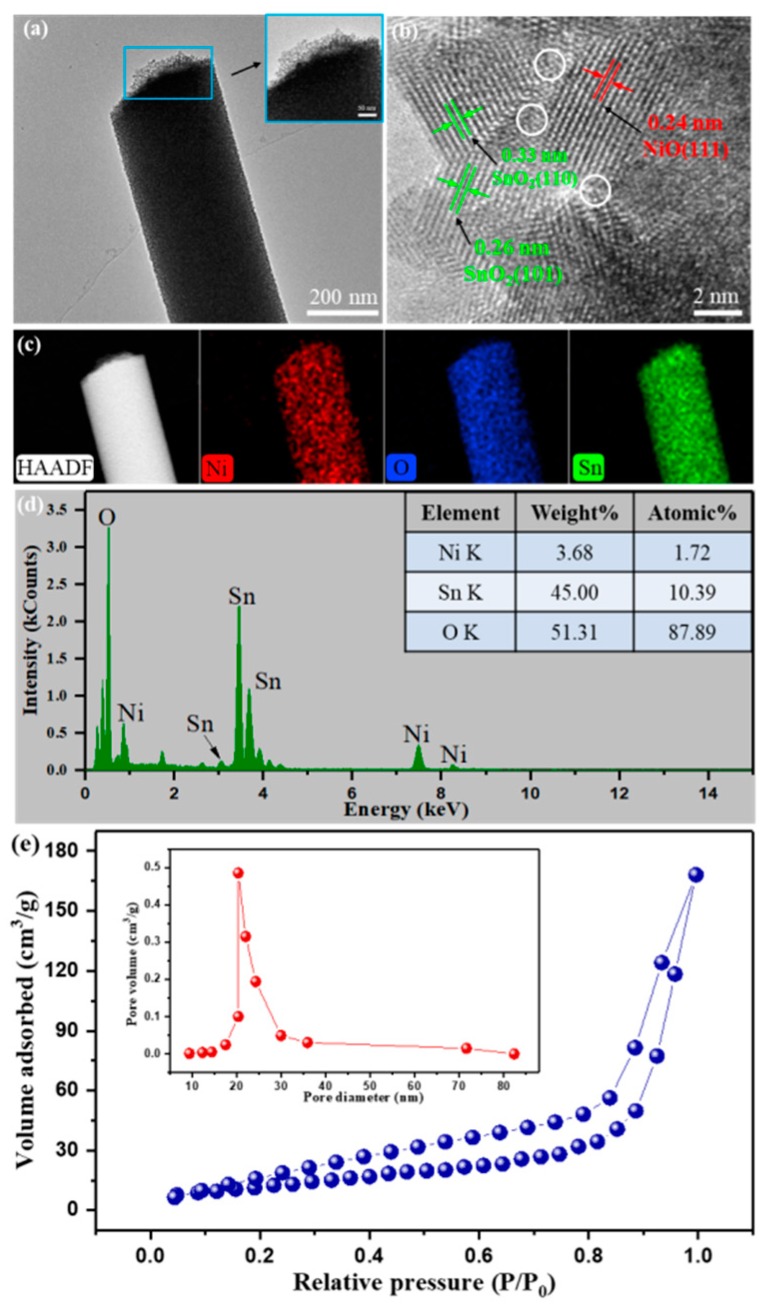
(**a**,**b**) High resolution transmission electron microscopy (HRTEM) images of the NiSn/4; (**c**) high angle annular dark field (HAADF) image and element mapping of a single NiSn/4 nanofiber; (**d**) The energy dispersive X-ray spectroscopy (EDS) spectrum of NiSn/4 nanocomposite. (**e**) The N_2_ adsorption-desorption isotherm and pore size distribution curve (inset) of the NiSn/4 nanofibers.

The morphological properties, nanostructures and chemical compositions along with contents of the NiSn/4 nanocomposite were studied by HRTEM, HAADF and EDS, respectively. As shown in Figure 4, the NiSn/4 nanofiber with a diameter about 200 nm was composed of a large number of stacked nanoparticles, and NiO and SnO_2_ particles cannot be clearly distinguished. This sample with unique porous nanostructure can provide more gas adsorption active sites and as a consequence may exhibit excellent gas-sensing performances. Obvious lattice fringes can be seen from Figure 4b, presenting a polycrystalline structure of the NiSn/4 sample. The marked lattice spacing of 0.33 nm and 0.26 nm shown no difference in the (110) and (101) planes of SnO_2_, respectively [38]. Besides, the lattice fringes with a spacing of 0.24 nm, which can be attributed to the NiO (111) plane [39]. The p-n heterojunctions exist between the SnO_2_ and NiO as marked by the white circle in Figure 4b. The difference in element weight can be reflected in various brightness of HADDF image, and element mapping can be used to analyze the distribution of sample elements. Based on these principles, we obtained the element composition and distribution results by means of characterization, as shown in Figure 4c. From Figure 4c, all images with uniform brightness and color, indicating that elements are evenly distributed in the nanofiber. EDS was used to further investigate the element composition and content of NiSn/4 nanocomposite. From the EDS spectrum (Figure 4d), we can see the presence of the elements O, Ni and Sn. The proportion of Ni atoms was calculated to be 14.2%, which was approximately the same as the experimental preset value (15%). The N_2_ adsorption-desorption isotherm and pore size distribution curve of the NiSn/4 nanofibers was shown in Figure 4e. From Figure 4e, we can find a distinct hysteresis loop, which indicates that the NiSn/4 nanofibers exhibit a large textural porosity. Moreover, the BET surface area of the NiSn/4 sample was calculated to be 43.57 m^2^/g. The corresponding pore size distribution was calculated by the BJH method [18]. The pore size distribution cure indicates that the relatively narrow pore size distribution centers are approximately 20.3 nm.

**Figure 5:**
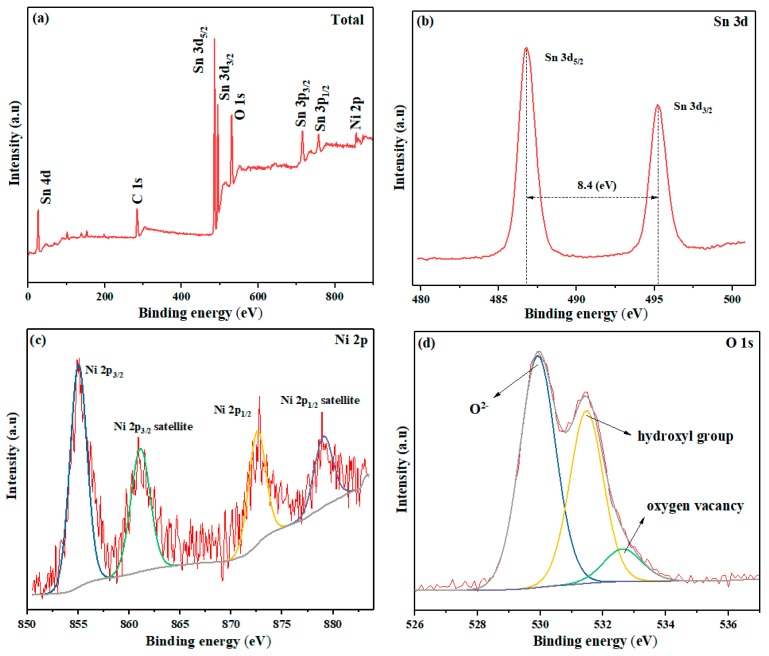
X-ray photoelectron spectroscopy (XPS) spectra of (**a**) total, (**b**) Sn, (**c**) Ni and (**d**) O for NiSn/4 sample.

The surface physical and chemical features of semiconductor nanomaterials, especially the oxygen defect content, play a significant part in gas-sensing properties of materials [40]. Therefore, we used XPS to study the elemental compositions and valence states of the NiSn/4 nanocomposite surface, and processed the peak fitting of the main elements. As presented in Figure 5a, the total spectrum of NiSn/4 sample surface mainly contains the elements of Sn, Ni, and O. The present of C peak with binging energy of 284.8 eV was introduced by the instrument itself for peak correction [41]. Figure 5b shows two peaks of Sn 3d_5/2_ (486.8 eV) and Sn 3d_3/2_ (495.2 eV) with binding energy difference of 8.4 eV, suggesting a normal chemical state of Sn^4+^ in the sample [42]. The divided high-resolution spectra of Ni 2p was fitted by Lorentz-Gauss Fitting, and shown in Figure 5c. Both the Ni 2p_3/2_ and Ni 2p_1/2_ are composed of the main peak and the satellite peak. The peaks of Ni 2p_3/2_ at 855.0 eV with its satellite peak (861.1 eV) and Ni 2p_1/2_ at 872.6 eV with its satellite peak (879.1 eV) indicated Ni^2+^ exists in the NiO/SnO_2_ composite [43]. From Figure 5d, the O1s peak is composed of three peaks located 529.9 eV, 531.5 eV and 532.6 eV, which were correspond for O^2−^, oxygen vacancies and hydroxyl groups, respectively [44].

### 3.2. Gas-Sensing Properties

**Figure 6:**
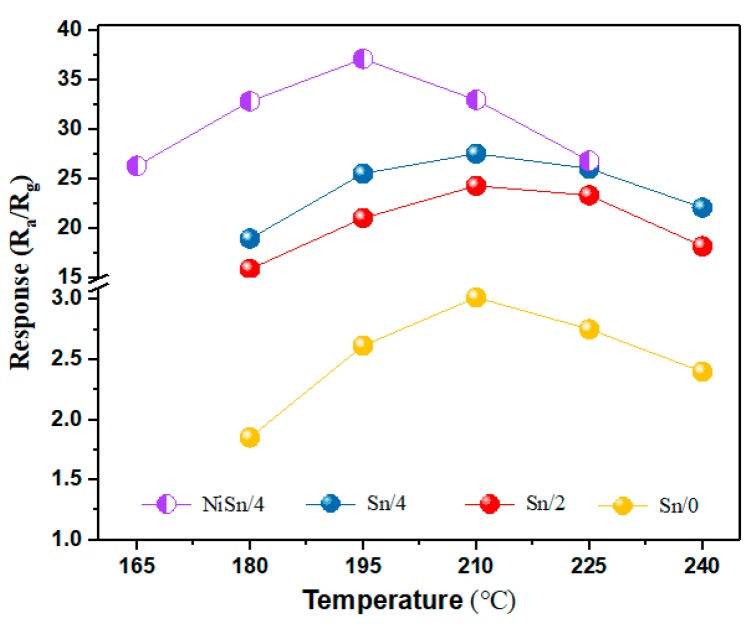
Gas response of the Sn/0, Sn/2, Sn/4 and NiSn/4 sensor devices to 100 ppm H_2_ under different operation temperatures.

Figure 6 shows the gas response of sensor devices to 100 ppm H_2_ at the operation temperatures ranging from 165 °C to 240 °C. From Figure 6, the gas response of sensors based on Sn/0, Sn/2, Sn/4 and NiSn/4 nanofibers initially increased with increasing temperature and reached the maximum values of 3.018, 24.293, 27.559 and 37.153, respectively. This process is mainly attributed to the fact that target gases can obtain more energy to surmount the energy barrier as working temperature increased, which promote the adsorption and reaction of the gas molecules and the surface of gas-sensing materials [45]. The gas response decreased with further temperature increases, which may be related to the enhancement of desorption process caused by the higher operation temperature. The optimal operation temperatures of Sn/0, Sn/2 and Sn/4 sensors were 210 °C, while the optimal operation temperature of NiSn/4 snesor was 195 °C. In addition, the gas response of the Sn/0 sensor is significantly lower than that of the sensor (Sn/2 and Sn/4) fabricated with carbonized nanofibers, which may due to the organic solvent contained in the nanofiber.

**Figure 7:**
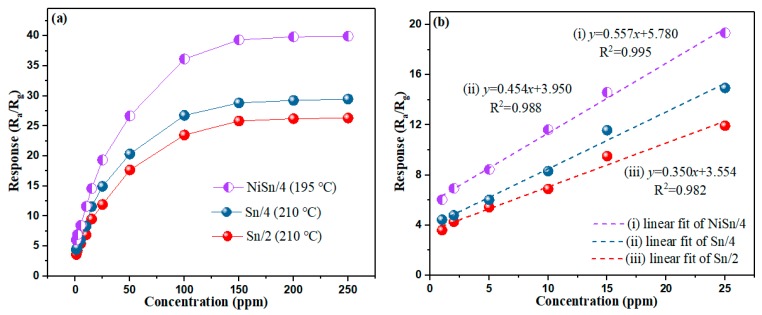
(**a**) Gas response of Sn/2, Sn/4 and NiSn/4 sensors to H_2_ with various concentrations at their optimal operation temperatures, respectively; (**b**) the linear fitting curves of the sensor devices at low H_2_ concentrations (1–25 ppm).

The gas response curves of Sn/2, Sn/4 and NiSn/4 sensors to H_2_ with various concentrations at their optimal operation temperatures are shown in Figure 7a. The gas responses of sensor devices increased with increasing H_2_ concentration up to 150 ppm, which is because of the more H_2_ molecules reacts with sensing- material, the more resistance of nanomaterial decreases. Additionally, the gas response of the NiSn/4 sensor device is higher than the other two sensors (Sn/2, Sn/4). This test results may be caused by the formation of p-n heterojunctions in the sample after the addition of NiO. We can also find from Figure 7a that the Sn/4 sample has an enhanced gas response than the Sn/2 sample, which may be related to the smaller grain size of the Sn/4 sample [36]. Figure 7b shows the corresponding linear fitting curves of the sensor devices at low H_2_ concentrations (1–25 ppm). The linear fitting results indicate that the synthesized samples have good linearity at low concentrations.

**Figure 8:**
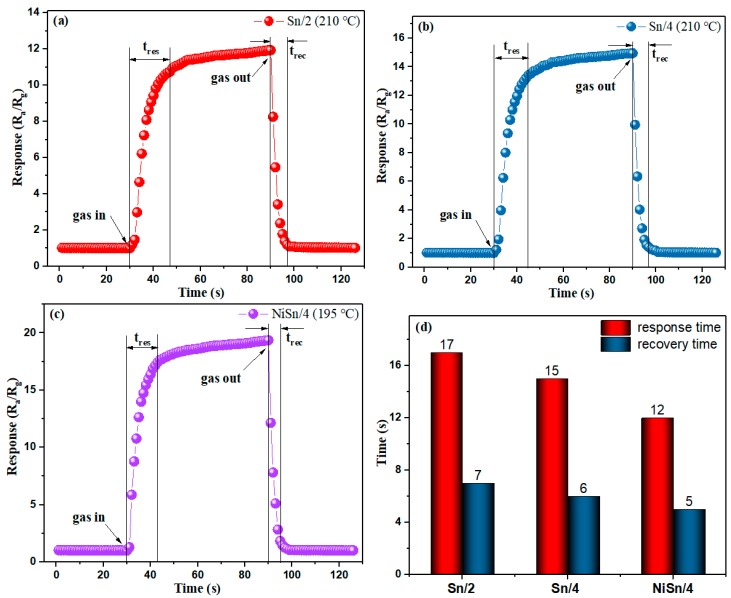
Response and recovery curves to 25 ppm H_2_ of (**a**) Sn/2, (**b**) Sn/4 and (**c**) NiSn/4 sensors, and (**d**) the corresponding response and recovery times.

It is widely acknowledged that the response and recovery characteristic is an important parameter of sensor devices. Thus, the response and recovery performances of Sn/2, Sn/4 and NiSn/4 sensors to 25 ppm H_2_ sensors was investigated as shown in Figure 8. It can be found from Figure 8a–c that the gas responses of all sensor devices show the same trend, that is, the gas responses of the Sn/2, Sn/4 and NiSn/4 sensors, gradually increase and tend to be stable at 11.934, 14.947 and 19.342, respectively, when H_2_ is injected, while they rapidly decrease when H_2_ is discharged. The corresponding response and recovery times of the Sn/2, Sn/4 and NiSn/4 sensors as shown in Figure 8d were calculated as approximately 17/7, 15/6 and 12/5 s, respectively. In addition, we can find from Figure 8d that the recovery time of the sensor is less than the response time. The possible reason could be that the unique porous nanostructure may provide an effective gas path for gas desorption, which allows H_2_ to be released quickly from the sensing materials [17].

**Figure 9:**
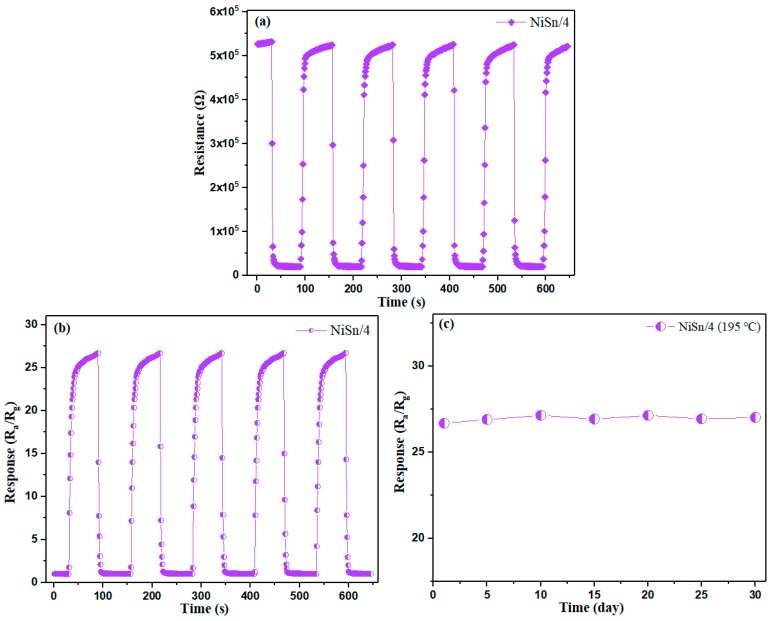
(**a**) The electric resistance properties of NiSn/4 sensor to 50 ppm H_2_ at 195 °C and (**b**) the corresponding dynamic gas-sensing curve. (**c**) The long stability of NiSn/4 sensor to 50 ppm H_2_ at 195 °C.

Figure 9a displays the resistance curve of the NiSn/4 sample toward 50 ppm H_2_ at 195 °C in five periods. As seen in Figure 9a, the resistance of the NiSn/4 sensor can be restored to the stable values of approximately 19.9 KΩ and 524.5 KΩ as the gas is introduced and discharged in five cycles. The corresponding dynamic gas-sensing curve is presented in Figure 9b. The gas response of the NiSn/4 sensor reaches the maximum value of approximately 26.674 with a standard deviation of 1.20% when the gas enters, and returns to the original value as the H_2_ exits, which confirms the excellent reproducibility of the gas-sensing performances. The long stability of NiSn/4 sensor to 50 ppm H_2_ at 195 °C is shown in Figure 9c. From Figure 9c, we can find that the gas response of NiSn/4 sensor demonstrates good reproducibility over the test period, which indicates the excellent stability of prepared NiSn/4 sensor.

In addition, the gas-sensing properties of some various nanostructures hydrogen sensors that have been reported in the last few years are listed in Table 1. Compared with those reported sensor, the present NiO/SnO_2_ sensor shows superior gas response, which indicates that our product is more likely to be used for H_2_ detection in real application.

### 3.3. Sensing Mechanism

In general, semiconducting sensing nanomaterials with interesting physical and chemical properties can detect various gases because of the adsorption gas molecules can exchange charge carriers with the semiconductor materials lead to the change of electrical resistance [55]. As exposing SnO_2_ (n-type with band gap of 3.6 eV) to air, its surface-adsorbed oxygen molecules will trap electrons from tin dioxide conduction band and ionize into diverse adsorbed oxygen (O^−^, O^2−^ and O_2_^−^) under the action of heat [32]. And the electric resistance of the sensing-material will be enhanced during this reaction process.

Whereas exposing SnO_2_ to H_2_, the chemically adsorbed oxygen will react with the H_2_ gas, and the free electrons will be released into the conduction band of SnO_2_, which ended up with reduced electrical resistance and increased gas response. Thus, the positive gas response (Ra/Rg) was measured. Compared with the Sn/2 sample, the improved gas sensitivity performances of the Sn/4 sample can be attributed to the fact that the Sn/4 with smaller grain size provides more active adsorption situs for gas adsorption and facilitates the reaction. That is to say, when exposing the Sn/4 nanofibers to H_2_, more electrons were released back to the sensing materials and the resistance value of Sn/4 in target gas (H_2_) was measured to be lower than that of the Sn/2 nanofibers, eventually resulting in the Sn/4 nanofibers exhibiting higher gas response.

**Figure 10:**
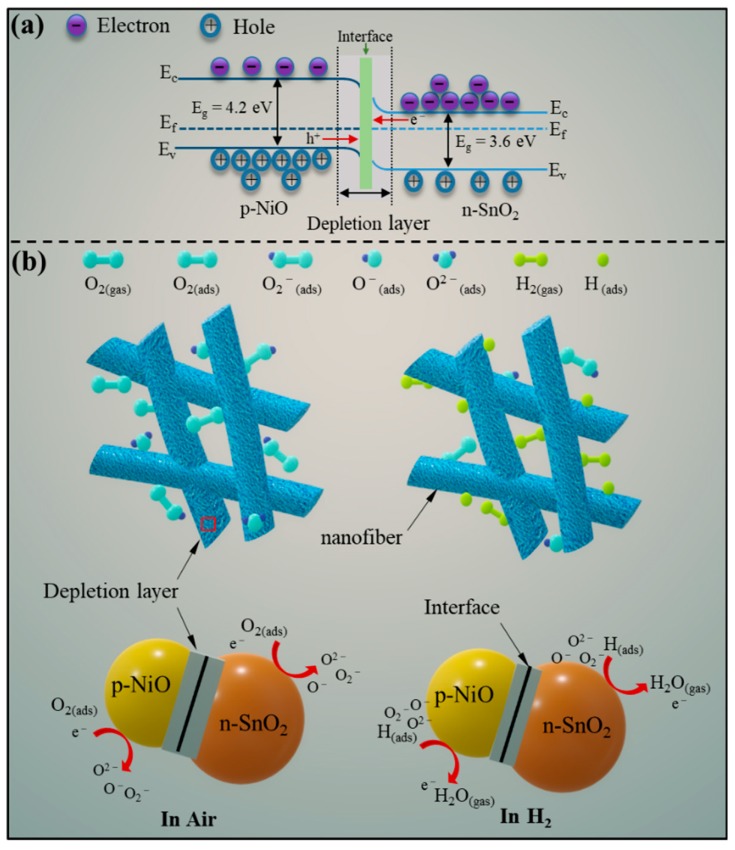
Schematic diagrams of (**a**) the energy band for p-n heterojunctions of NiO/SnO_2_ and (**b**) the proposed H_2_ sensing mechanism for the NiO/SnO_2_ nanocomposite.

The enhanced gas-sensing property of the NiO/SnO_2_ sensor is attributable to the formation of p-n heterojunctions at the interfaces between NiO and SnO_2_. As we all know, NiO as a typical p-type semiconductor with the band gap of 4.2 eV shows conductivity by holes [56]. When NiO and SnO_2_ make contact with each other, the electrons will transfer from SnO_2_ to NiO, while the holes transfer will be reversed due to the difference in concentration of the charge carriers [57]. The semiconductor band is significantly bent until the Fermi level is equal as shown in Figure 10a, resulting in the formation of a self-built electric field and p-n heterojunctions at the interface [58]. This heterojunction effect leads to the formation of a depletion layer.

When the NiO/SnO_2_ nanocomposite is exposed to air, as shown in Figure 10b, oxygen molecules can absorb on the surface of the sensing material and trap electrons in the NiO/SnO_2_ sample to form adsorbed oxygen. The electric resistance in air (Ra) of NiO/SnO_2_ sample is higher than that of the pure SnO_2_ sample due to the existence of depletion layer [59]. When H_2_ was injected, the absorbed oxygen on the surface of NiO/SnO_2_ nanofiber react with target molecules and release electrons back to the SnO_2_ conduction band and combine with the holes of NiO. This process will lead to the depletion layer significantly narrows and the electric resistance of the NiO/SnO_2_ nanocomposites obviously decreases [32]. Therefore, according to the definition of gas response (Ra/Rg), the enhanced gas response of the sensor was measured due to the variation of resistance [60]. In short, as-prepared NiO/SnO_2_ composite nanofibers with p-n heterojunctions exhibit enhanced gas-sensing properties to hydrogen.

## 4. Conclusions

In summary, the porous sensing materials of SnO_2_-based nanofibers have successfully synthesized via electrospinning method along with carbonization process in various heating rates. The results of characterization indicate that carbonization process can make the pristine nanofibers form porous nanostructure. Gas-sensing investigations prove that various ageing heating rates can cause changes in the micro grain size of SnO_2_ nanofibers, leading to differences in gas-sensing properties. In addition, the NiO/SnO_2_ nanocomposites based sensor shows improved gas-sensing properties to H_2_ by comparing with the pure one. Specifically, the optimum working temperatures of the NiO/SnO_2_ (NiSn/4) and pure SnO_2_ (Sn/4) sensors were tested to be about 210 °C and 195 °C, respectively. The corresponding gas responses to 100 ppm H_2_ under their optimum working temperatures reached 37.153 and 27.559, respectively. Moreover, the superior hydrogen sensing properties of the NiO/SnO_2_ sensor with good low concentration linearity, short response-recovery time and excellent reproducibility are also obtained. This work suggests that the NiO/SnO_2_ nanocomposites with unique porous and p–n heterojunctions would make it as a potential candidate for superior-performance sensing of gases.

## Figures and Tables

**Table 1:** Comparison of the H_2_ sensing properties with different sensors.

Materials	H_2_ (ppm)	Optimal Temperature (°C)	Response (Ra/Rg)	Year	Reference
rGO/ZnO composite	200	150	3.5	2014	[46]
Pd/SnO_2_ thin film	250	300	28.0	2016	[47]
Nb_2_O_5_-NiO nanocomposite	500	R.T.	1.68	2017	[48]
WO_3_-ZnO nanowire	2000	200	12.6	2019	[49]
Mg-In_2_O_3_ nanotubes	100	150	1.55	2015	[50]
Si nanowires	50	100	17.1	2018	[51]
Pt-SnO_2_ hollow microspheres	200	50	21.0	2018	[52]
NiO/SnO_2_ nanocomposite	100	320	13.6	2010	[53]
NiO/SnO_2_ nanospheres	50	325	27.84	2015	[54]
NiO/SnO_2_ nanofibers	100	195	37.15		This work
